# The Treg-Specific Demethylated Region Stabilizes *Foxp3* Expression Independently of NF-κB Signaling

**DOI:** 10.1371/journal.pone.0088318

**Published:** 2014-02-05

**Authors:** Lisa Schreiber, Beate Pietzsch, Stefan Floess, Carla Farah, Lothar Jänsch, Ingo Schmitz, Jochen Huehn

**Affiliations:** 1 Department Experimental Immunology, Helmholtz Centre for Infection Research, Braunschweig, Germany; 2 Research Group Cellular Proteomics, Helmholtz Centre for Infection Research, Braunschweig, Germany; 3 Research Group Systems-oriented Immunology and Inflammation Research, Helmholtz Centre for Infection Research, Braunschweig, Germany; 4 Institute of Molecular and Clinical Immunology, Otto-von-Guericke-University, Magdeburg, Germany; Institut Pasteur, France

## Abstract

Regulatory T cells (Tregs) obtain immunosuppressive capacity by the upregulation of *forkhead box protein 3* (*Foxp3*), and persistent expression of this transcription factor is required to maintain their immune regulatory function and ensure immune homeostasis. Stable *Foxp3* expression is achieved through epigenetic modification of the Treg-specific demethylated region (TSDR), an evolutionarily conserved non-coding element within the *Foxp3* gene locus. Here, we present molecular data suggesting that TSDR enhancer activity is restricted to T cells and cannot be induced in other immune cells such as macrophages or B cells. Since NF-κB signaling has been reported to be instrumental to induce *Foxp3* expression during Treg development, we analyzed how NF-κB factors are involved in the molecular regulation of the TSDR. Unexpectedly, we neither observed transcriptional activity of a previously postulated NF-κB binding site within the TSDR nor did the entire TSDR show any transcriptional responsiveness to NF-κB activation at all. Finally, the NF-κB subunit c-Rel revealed to be dispensable for epigenetic imprinting of sustained *Foxp3* expression by TSDR demethylation. In conclusion, we show that NF-κB signaling is not substantially involved in TSDR-mediated stabilization of *Foxp3* expression in Tregs.

## Introduction

Regulatory T cells (Tregs) are cellular mediators of immunological tolerance as they possess the capacity to suppress various types of immune responses against self and non-self antigens [1]. The transcription factor *forkhead box protein 3* (*Foxp3*) is specifically expressed in Tregs and is essential for the development of their immunosuppressive properties [2], [3]. After induction during Treg development, continued expression of *Foxp3* is imperative for the maintenance of the cells’ suppressive phenotype [4]. Foxp3^+^ Tregs bear a curative potential and are considered for various therapeutic applications [5], however, instability of *Foxp3* expression and concomitant acquirement of proinflammatory properties are major obstacles. Stable *Foxp3* expression is accompanied by epigenetic modulation of the Treg-specific demethylated region (TSDR), a CpG-rich, non-coding sequence within the first intron of the *Foxp3* gene locus [6]. The TSDR is demethylated only in Tregs stably expressing *Foxp3* but is fully methylated in CD4^+^ conventional T cells (Tconv) and in *in vitro* generated Tregs only transiently expressing *Foxp3* [7]–[9]. Moreover, transcriptional enhancer activity of the TSDR in an *in vitro* reporter assay is essentially determined by its methylation status [10]. It is completely inactive in its methylated state, but as soon as the TSDR is demethylated transcription factors such as Ets-1 and Creb can bind to the TSDR [8], [10], [11] and switch on its transcriptional activity, most likely in cooperation with other transcription factors that have been demonstrated to occupy the TSDR, e.g. Stat-5 and Runx1/3 [12]. Despite the critical role of the TSDR for stabilization of *Foxp3* expression, the molecular players participating in its transcriptional regulation are only incompletely understood. Elucidating the underlying molecular mechanisms may open up new approaches to modulate stability of *Foxp3* expression, which is of outmost importance for the therapeutic application of Tregs in clinical settings.

Recently, NF-κB transcription factors, which are important, inducible regulators of innate and adaptive immunity [13], have been shown to be involved in Treg development [14]. In mammals, the NF-κB protein family consists of five members: p65 (RelA), c-Rel, RelB, p50 and p52, all of which share a structural motif known as Rel homology domain that is critical for homo- or hetero-dimerization and DNA binding. Without an appropriate stimulus, NF-κB proteins are trapped in the cytoplasm by inhibitors of NF-κB (IκB) proteins, which shield the nuclear localization sequence of NF-κB, thereby preventing their translocation into the nucleus. Similarly, p100 and p105, which are the unprocessed precursor proteins of p52 and p50, respectively, function as IκB (reviewed in [15]). In T cells, ligation of the T cell receptor (TCR) complex induces the canonical NF-κB signaling pathway, which integrates the activation of the IκB kinase (IKK) complex [16]. The two catalytic subunits IKKα and IKKβ, in cooperation with the scaffold protein IKKγ, can mediate IκB phosphorylation, which is followed by ubiquitination and degradation of IκB, thus releasing NF-κB proteins and permitting nuclear localization of transcriptionally active p65/p50 and p50/c-Rel heterodimers [15]. IKKβ-mediated IκBα degradation is regarded as the central step in canonical NF-κB activation in T cells [15]. In contrast, IKKα activity is crucial for the activation of the non-canonical NF-κB pathway, which targets activation of p52/RelB heterodimers [17].

Many of the TCR/NF-κB signaling mediators contribute to normal Treg development, namely IKKβ, protein kinase C-θ and components of the CBM (Carma-1/Bcl-10/Malt-1; Card [caspase-recruitment domain]-Maguk [membrane-associated guanylate kinase] protein-1, B cell lymphoma-10, mucosa-associated lymphoid tissue lymphoma translocation gene-1) complex, which functions upstream of IKKβ activation, as well as IκBα, the NF-κB subunit c-Rel [18]–[27] and the atypical IκB protein IkB_NS_ [28]. Importantly, it was shown that NF-κB can directly target *Foxp3* gene expression [25], [28]–[31], and c-Rel binding to the *Foxp3* promoter as well as to the recently described pioneer element [30] is crucial for initiating *Foxp3* expression during Treg development [32]. However, the contribution of NF-κB to the transcriptional regulation of the TSDR is discussed more controversially [32]. c-Rel binding to the TSDR could be detected in primary Tconv [25], and we have recently reported a potential NF-κB binding site within the TSDR that is critical for full TSDR enhancer activity and that is identical to the NF-κB binding site postulated by Long *et al*. [10], [25]. In contrast, Ruan *et al*. detected binding of c-Rel and p65 exclusively to the *Foxp3* promoter in TCR-stimulated T cells, whereas no binding to the TSDR could be observed in the same cells [29]. Therefore, the contribution of NF-κB to the transcriptional regulation of the TSDR remains obscure and it is not clear whether NF-κB is required for the stabilization of *Foxp3* expression.

In the present study, we could demonstrate that the transcriptional enhancer activity of the TSDR is entirely dependent on T cell-specific signals and is not inducible in other immune cells. Moreover, blocking NF-κB signaling pathways did not significantly influence TSDR enhancer activity, indicating that the TSDR functions in an NF-κB-independent manner. Finally, stable *Foxp3* expression and epigenetic modifications of the TSDR were observed in c-Rel-deficient Tregs. In summary, these data suggest that NF-κB factors are largely dispensable for TSDR-mediated stabilization of *Foxp3* expression and epigenetic remodeling of the *Foxp3* locus.

## Results

### TSDR Enhancer Activity Requires T cell-specific Signals

**Figure 1:**
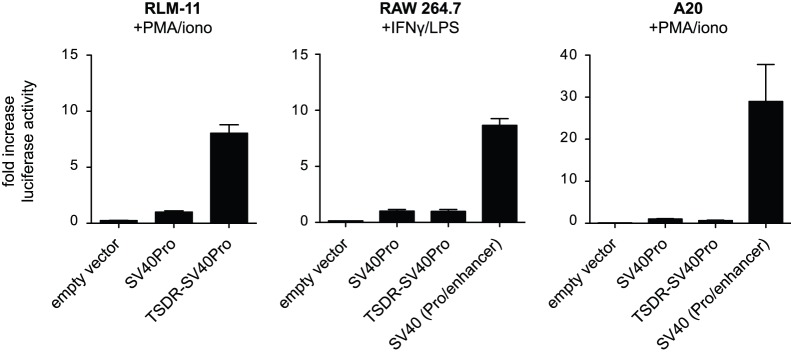
B cells and macrophages fail to induce transcriptional enhancer activity of the TSDR. Dual luciferase assays were performed after transfecting reporter plasmids carrying the indicated inserts or an empty pGL3 vector (EV) into RLM-11 cells (T cell line), A20 cells (B cell line) or RAW 264.7 cells (macrophage cell line). Three hrs (RLM-11, A20) or 20 hrs (RAW 264.7) after transfection, cells were stimulated for 16 hrs with PMA/iono (RLM-11, A20) or for 14 hrs with LPS/IFN-γ (RAW 264.7), followed by measurement of luciferase activities (mean±SD, n = 3). Data are representative of two to four independent experiments.

Compelling evidence suggests that the TSDR acts as a transcriptional stabilizer of *Foxp3* expression in Tregs [6]. In combination with a promoter, the TSDR possesses transcriptional enhancer activity in luciferase reporter assays, which have therefore been a common approach to analyze its transcriptional regulation [7], [10], [25], [30]. In the present literature, TSDR enhancer activity has been demonstrated in T cell lines as well as in primary T cells, and this was dependent on the activation of several transcription factors that have usually been triggered by the application of phorbol-12-myristate-13-acetate (PMA) and ionomycin (PMA/iono) mimicking the TCR stimulus (reviewed in [12]). However, it is not known whether the TSDR can be activated in non-T cells. In order to determine whether other cell types can deliver the molecular requirements to stimulate TSDR activity, we assessed TSDR enhancer activity in the B lymphoma cell line A20 and the macrophage cell line RAW 264.7 and performed luciferase reporter assays using plasmids encoding luciferase under the control of the TSDR and a promoter. Initially, we tested TSDR enhancer activity in combination with the endogenous *Foxp3* promoter. Although the TSDR-*Foxp3* promoter construct was sufficient to drive luciferase expression in the T lymphoma cell line RLM-11 [10], weak (A20) or no (RAW 264.7) luciferase activity was observed in A20 and RAW 264.7 cells upon stimulation with PMA/iono (data not shown). We therefore repeated luciferase assays using plasmids integrating the SV40 promoter in combination with the TSDR and measured transcriptional activity in RLM-11 cells, A20 cells and RAW 264.7 cells (Fig. 1). The SV40 promoter was transcriptionally active in all cells and gave rise to increased luciferase activities as compared to empty vector controls upon PMA/iono stimulation (A20 and RLM-11) or interferon-γ (IFN-γ)/lipopolysaccharide (LPS) stimulation (RAW 264.7). As expected, the TSDR was able to augment this activity in RLM-11 cells. In contrast, A20 and RAW 264.7 cells displayed no enhanced luciferase activity in the presence of the TSDR (Fig. 1). However, A20 and RAW 264.7 cells showed increased luciferase activity when reporter vectors containing an SV40 enhancer were used. Viewed as a whole, these data implicate that the TSDR depends on T cell-specific signals and cannot be activated in other immune cell types.

### A Postulated NF-κB Binding Site within the TSDR is not Responsive to NF-κB Activation in T cells

Comprehensive research has provided evidence that intrinsic NF-κB signaling is crucial for the development of thymus-derived Tregs [14]. Several analyses indicate that NF-κB directly participates in the transcriptional regulation of *Foxp3* expression [14], [32]. In this study, investigations focused on the role of NF-κB signaling in the regulation of TSDR enhancer activity.

**Figure 2:**
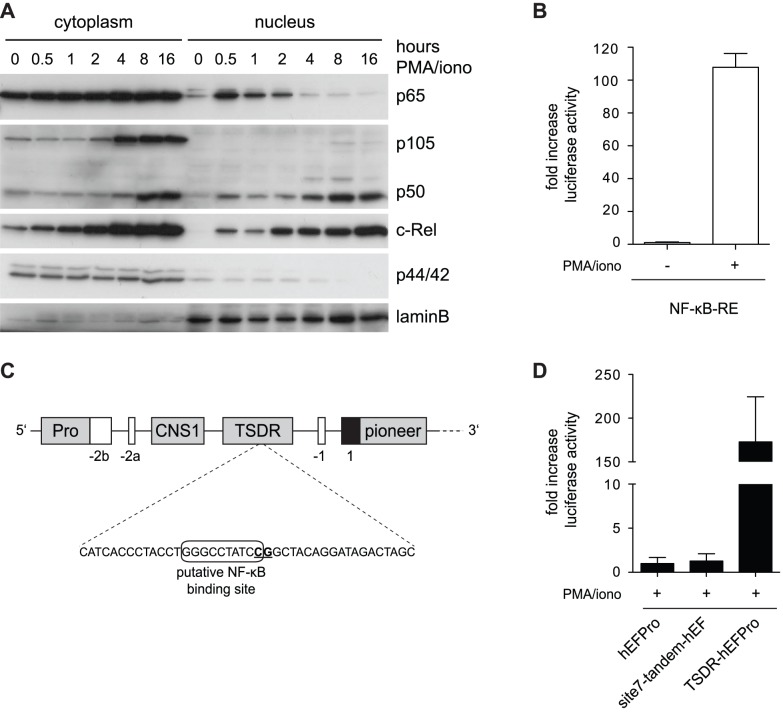
The postulated NF-κB binding site of the TSDR is not transcriptionally responsive to activated NF-κB. (**A**) RLM-11 cells were stimulated with PMA/iono for indicated time periods and applied to subcellular fractionation. Nuclear and cytoplasmic extracts were analyzed by Western blotting using the indicated antibodies. p44/42 and lamin B served as loading and purity controls for cytoplasmic and nuclear fractions, respectively. (**B**) A luciferase reporter plasmid integrating the NF-κB-RE was transfected into RLM-11 cells and dual luciferase assays were performed in triplicates as described in Figure 1. Mean luciferase activity is shown as fold increase to unstimulated control. Results are representative of four independent experiments. (**C**) A schematic view on the first part of the *Foxp3* gene locus is depicted. White boxes indicate untranslated exons, the first translated exon is indicated in black. Evolutionary conserved non-coding sequences (CNS) are indicated in grey. The distended region of the TSDR includes the previously described NF-κB binding site (black frame), which is flanked by the seventh CpG motif (underlined) of the TSDR. (**D**) A tandem of five repetitive sequences of the putative NF-κB binding site was inserted into the pCpGL luciferase reporter plasmid upstream of the *EF* promoter (tandem-EFPro). Dual luciferase assays were performed as described in (B) using pCpGL-TSDR-EFPro as a positive control. Data represent one out of two independent experiments.

First, we aimed to confirm the activation of NF-κB transcription factors in RLM-11 cells, which we intended to use for subsequent luciferase experiments. To this end, RLM-11 cells were stimulated with PMA/iono for increasing time periods and subcellular localization of NF-κB was analyzed by Western blotting. Expression of NF-κB proteins p105 and c-Rel increased in the cytoplasm upon stimulation and the activated subunits p50, p65 and c-Rel translocated to the nucleus (Fig. 2a and S1). Similar results have been obtained in a time course of NF-κB activation in primary murine CD4^+^ T lymphocytes [28], indicating similar kinetics of NF-κB activation in RLM-11 cells and primary T cells. Next, transcriptional activity of NF-κB was tested by means of a luciferase assay using the NF-κB-responsive element (NF-κB-RE), a sequence of five repetitive NF-κB binding sites, which drives luciferase expression upon NF-κB activation with the help of a minimal promoter element (TATA box). Upon transfection of RLM-11 cells, PMA/iono stimulation potently induced activation of the NF-κB-RE (Fig. 2b). Thus, in accordance with NF-κB nuclear translocation, NF-κB is transcriptionally active in RLM-11 cells.

A putative binding site for NF-κB within the TSDR (Fig. 2c) had been described previously to be critically required for the transcriptional enhancer activity of the TSDR [10], [25]. However, a final proof that NF-κB binds to this critical site in order to promote TSDR enhancer activity is lacking so far. In order to test whether this sequence comprises NF-κB-mediated transcriptional activity, five repetitive sequences of this putative NF-κB binding site (tandem) were introduced into a luciferase construct containing the *human elongation factor 1* (*EF*) promoter and luciferase assays were performed in RLM-11 cells (Fig. 2d). Despite strongly enhanced *EF* promoter activity when combined with the full TSDR, repetitive sequences of the putative NF-κB binding site alone were not sufficient to enhance *EF* promoter activity, suggesting that this sequence does not function as a transcriptional responder of NF-κB activation.

### Activation of NF-κB Signaling Mediators is Largely Dispensable for TSDR Enhancer Activity

**Figure 3:**
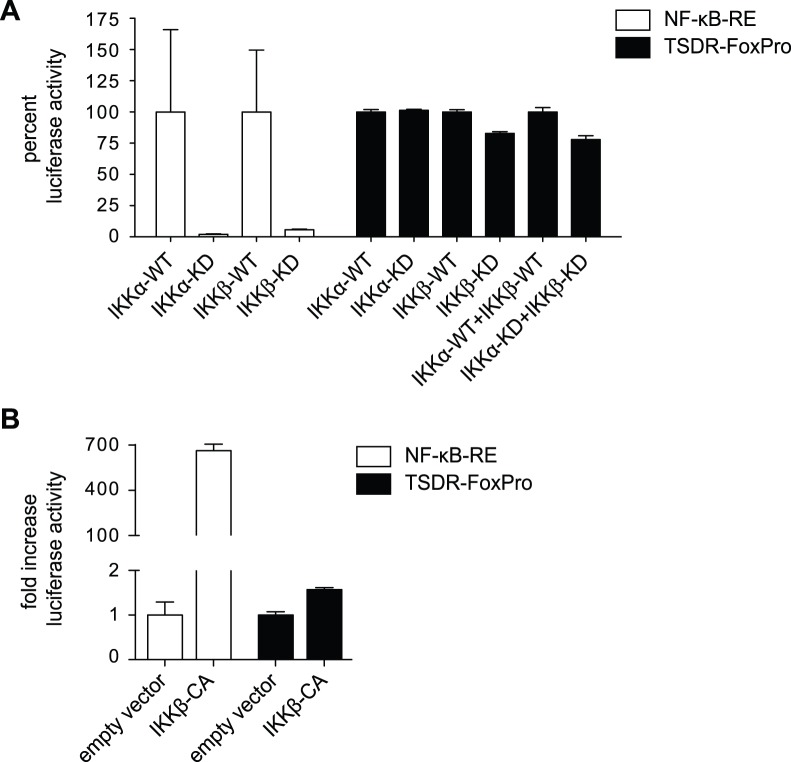
Kinase activity of IKKα and IKKβ is largely dispensable for TSDR enhancer activity. (**A**) Luciferase plasmids encoding NF-κB-RE or TSDR-FoxPro were co-transfected with plasmids encoding kinase dead (KD) or wild-type (WT) forms of IκB kinase α and β (IKKα and IKKβ) into RLM-11 cells. Cells were cultured for one day allowing efficient protein expression before cells were stimulated overnight with PMA/iono and dual luciferase assays were performed. Luciferase activities are given as percent of luciferase activity of WT samples and standard deviations were calculated from three replicates. (**B**) Dual luciferase assays as described in (A) were performed co-transfecting the indicated luciferase constructs with a plasmid encoding the constitutively active form of IKKβ (IKK-CA) or empty vector as control (mean±SD, n = 3). One representative out of three independent experiments is shown.

NF-κB binding to the TSDR has been observed by chromatin immunoprecipitations [25] and thus, it was still possible that NF-κB signaling was important to obtain full TSDR transcriptional activity mediated by yet unidentified NF-κB binding sites. To address the general impact of NF-κB signaling on the TSDR, functional involvement of IκB kinases (IKK) was analyzed. To this end, TSDR enhancer activity was measured in a luciferase assay in RLM-11 cells in which kinase dead (KD) mutants of IKKα or IKKβ were overexpressed (Fig. 3a). Wild-type (WT) IKKs served as controls and functionality of the IKK-KD mutants was proven with luciferase constructs carrying the NF-κB-RE. Whereas overexpression of IKKα-KD or IKKβ-KD drastically reduced transcriptional activity of the NF-κB-RE, no comparable effect was observed for the TSDR albeit overexpression of IKKβ-KD moderately reduced TSDR enhancer activity to about 80% (Fig. 3a). Furthermore, combined overexpression of IKKα-KD and IKKβ-KD had no synergistic capacity to further minimize TSDR enhancer activity (Fig. 3a). In a complementary approach, constitutively active IKKβ (IKKβ-CA) [33] was tested for its ability to enhance TSDR activity. To this end, luciferase activity of the TSDR was tested in RLM-11 cells that had been co-transfected with IKK-CA or an empty vector as control (Fig. 3b). Functionality of IKK-CA was verified in a luciferase assay using the NF-κB-RE, giving rise to a more than 600-fold increase of luciferase activity upon overexpression of IKKβ-CA. However, only moderate induction of TSDR enhancer activity was observed upon IKK-CA overexpression.

**Figure 4:**
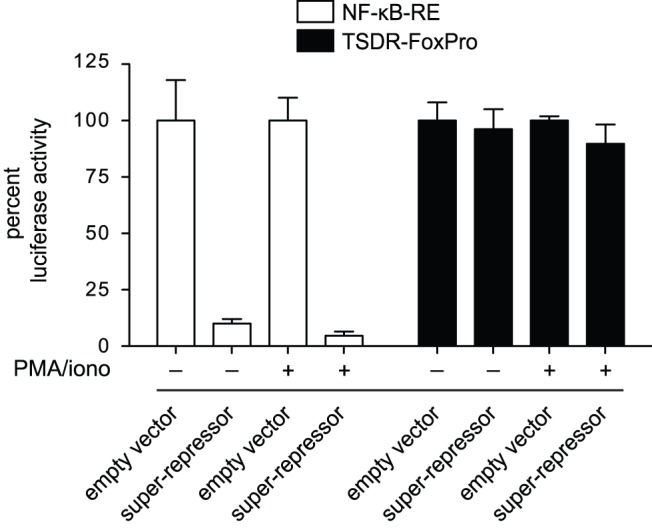
Degradation of IκBα is not required for TSDR enhancer activity. Luciferase plasmids integrating either the NF-κB-RE or TSDR-FoxPro were co-transfected with either an empty vector or with a vector encoding the super-repressor, a non-degradable form of IκBα, into RLM-11 cells. Dual luciferase assays were performed as described in Figure 1 and unstimulated cells served as controls. Luciferase activities are shown as percent of empty vector controls and standard deviations of performed triplicates are shown. One representative experiment out of at least two independent experiments is depicted.

Kinase activity of IKKβ is required to phosphorylate IκBα to target it for degradation. However, it has been shown that IKK additionally phosphorylates other proteins [15]. As IKKβ moderately influenced TSDR activity, the role of canonical NF-κB signaling in the regulation of TSDR activity was further investigated. For this purpose, luciferase assays were performed in RLM-11 cells overexpressing a phosphorylation- and degradation-resistant mutant form of IκBα, the degradation of which is usually regarded to be a central step during canonical NF-κB signaling. This mutant containing serine-to-alanine changes at amino acid residues 32 and 36 is commonly referred to as NF-κB super-repressor [34]. Luciferase activities of the TSDR-*Foxp3* promoter construct and the NF-κB-RE were measured in the presence or absence of the super-repressor (Fig. 4). Overexpression of the super-repressor drastically reduced activity of the NF-κB-RE down to levels that were similar to those of unstimulated cells (Fig. 4). In contrast, transcriptional enhancer activity of the TSDR was unaffected upon overexpression of the super-repressor at various time points after stimulation, indicating that degradation of IκBα was not required to stimulate full TSDR enhancer activity (Fig. 4 and S2). In summary, our results propose that activation of canonical as well as non-canonical NF-κB signaling is dispensable for TSDR enhancer activity.

### c-Rel is not Required for TSDR Demethylation and Stable *Foxp3* Expression

**Figure 5:**
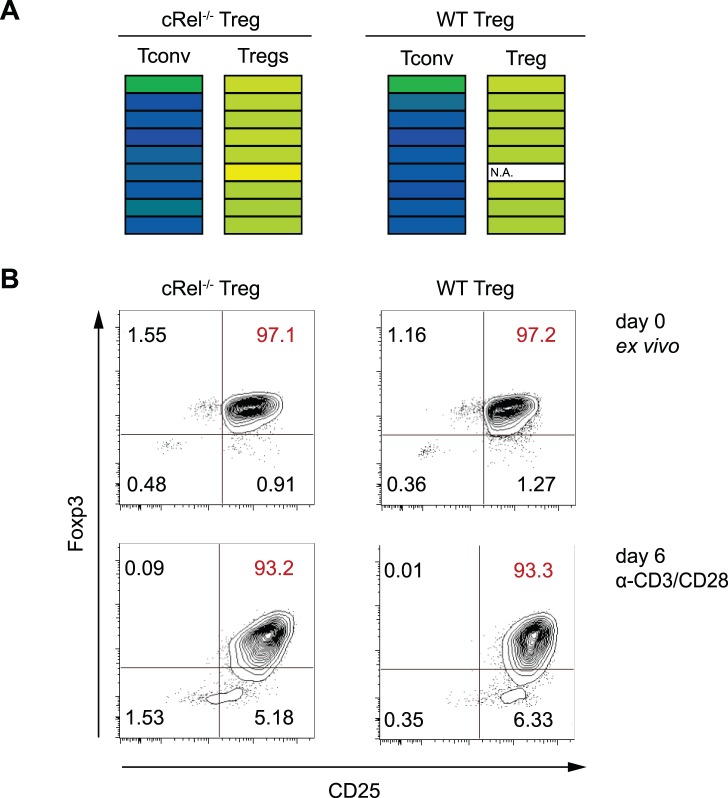
c-Rel^−/−^ Tregs show a stable phenotype. (**A**) CD4^+^CD25^hi^ Tregs and CD4^+^CD25^−^ Tconv were isolated from wild-type (WT) or c-Rel^−/−^ mice. Genomic DNA was isolated and subjected to bisulfite sequencing in order to determine the methylation status of CpG dinucleotides within the TSDR. (**B**) CD4^+^CD8^−^CD62L^hi^CD25^hi^ Tregs from spleen and lymph nodes of c-Rel^−/−^ or WT mice were sorted and an aliquot was analyzed for Foxp3 expression by flow cytometry (top panel). Cells were cultured in the presence of IL-2 and stimulated by plate-bound α-CD3/CD28 for six days followed by flow cytometric analysis of Foxp3 expression. Cells depicted were pregated to viable CD4^+^ T cells. Results represent one out of two independent experiments.

The NF-κB subunit c-Rel was described to be the most critical factor of all NF-κB proteins during the thymic development of Tregs [21], [26], [35]. c-Rel has been shown to bind to the TSDR in Tconv [25], however, whether c-Rel plays a functional role in the TSDR-mediated stabilization of *Foxp3* expression has not been addressed yet. Ample evidence has accumulated suggesting that DNA demethylation at the TSDR is indispensable for the maintenance of *Foxp3* expression [6]. Furthermore, c-Rel has been described to encompass chromatin-remodeling properties [36], and it was therefore conceivable that c-Rel was involved in TSDR demethylation. To address this question, Tregs and Tconv were isolated from c-Rel^−/−^ mice and WT controls. Cells were analyzed for their DNA methylation status at the TSDR (Fig. 5a). As expected, Tconv from both WT as well as c-Rel^−/−^ mice were completely methylated at each of the nine CpG motifs analyzed. Importantly, Tregs from c-Rel^−/−^ and WT mice revealed comparable levels of TSDR demethylation, demonstrating that c-Rel was dispensable for establishing this epigenetic mark. Finally, c-Rel^−/−^ Tregs were analyzed for their capability to maintain *Foxp3* expression upon activation and proliferation. Thereto, c-Rel^−/−^ or WT Tregs were isolated to high purity, cultured for six days using CD3/CD28 stimulation in the presence of IL-2 and analyzed for stability of Foxp3 expression at the end of the culture. A comparably high frequency of WT and c-Rel^−/−^ Tregs maintained *Foxp3* expression, indicating that c-Rel was not required for the stabilization of *Foxp3* expression (Fig. 5b).

In summary, we could show that the canonical NF-κB signaling pathway is largely dispensable for the control of TSDR enhancer activity and that the transcription factor c-Rel is not involved in TSDR-mediated stabilization of *Foxp3* expression.

## Discussion

Stable *Foxp3* expression is indispensable for the maintenance of the established transcriptional program and suppressive capacity of Tregs. In Tregs, *Foxp3* expression is epigenetically imprinted and occurs via demethylation of a highly conserved CpG-rich region within the *Foxp3* locus, designated TSDR [9], [37], [38]. Since the demethylated TSDR presents an epigenetic mark, which is unique to Tregs, it can clearly distinguish these cells from other cell types [39]. On a functional level, the TSDR stabilizes *Foxp3* expression and thereby determines Treg lineage stability [6], [40]. However, the molecular mechanisms that control TSDR activity are only incompletely understood. Here, we could show that the transcriptional enhancer activity of the TSDR is entirely dependent on T cell-specific signals and can neither be induced in B cells nor macrophages. Furthermore, NF-κB signaling pathways did not significantly influence TSDR activity, suggesting that NF-κB factors are largely dispensable for TSDR-mediated stabilization of *Foxp3* expression.

Various studies have demonstrated that the TSDR accommodates transcriptional enhancer activity in human and murine primary and immortalized T cells and, importantly, that methylation of the TSDR completely abrogates this transcriptional activity [7], [8], [30], [41]. On the contrary, forced demethylation of the TSDR by the application of the demethylating drug 5-azacytidine could artificially induce stable *Foxp3* expression in Tconv [7]. Interestingly, this is also true for some non-CD4^+^ T cells: *Foxp3* expression was observed in primary CD8^+^ T cells lacking DNA methyltransferase-1 [42], and both, natural killer cells and CD8^+^ T cells exhibited *Foxp3* gene expression when activated and cultured in the presence of 5-azacytidine [8], [43].

These non-CD4^+^ T cells share essential signaling molecules with CD4^+^ T cells, including the ζ-chain and the IL-2 receptor β-chain CD122 as well as the downstream transcription factors Creb and Stat-5 [44], [45], which were shown to bind to and transactivate the TSDR [8], [46]. Thus, it is tempting to speculate that these signaling pathways are key control elements of the *Foxp3* locus in Tregs and that solely the methylation status of the TSDR prevents *Foxp3* expression in CD8^+^ T cells and natural killer cells.

However, the methylation status of the TSDR was not the only determinant controlling *Foxp3* gene activity. We could provide evidence that the transcriptional activity of the TSDR also depends on cell-type-specific signaling pathways. Whereas the TSDR can be activated in T cell lines such as RLM-11 (this study) or EL-4 [30], TSDR activity could not be detected in B cell or macrophage cell lines upon stimulation with PMA/iono or LPS/IFN-γ. Hence, even though the TSDR was fully demethylated in the luciferase reporter assays in the present study, PMA/iono is only capable of stimulating TSDR activity in T cells, but not in B cells or macrophages, indicating that mere triggering of calcium influx (ionomycin) and protein kinase C activity (PMA) is not sufficient to activate the TSDR. Rather, B cells and macrophages seem to lack expression of essential signaling mediators or transcription factors that are required to promote full TSDR activity. Hence, the dependency on T-cell-specific signaling pathways may ensure that (stable) *Foxp3* expression is permitted only in T cells.

In recent years numerous studies have focused on the identification of transcription factors that are involved in the regulation of *Foxp3* gene expression [12]. Among them the NF-κB family of transcription factors has been shown to be crucial for Treg development. The NF-κB signaling pathway is considered to transduce signals emanating from the TCR in order to induce *Foxp3* expression during thymic Treg development [14], [32] and direct binding of NF-κB to the *Foxp3* gene locus has frequently been reported [25], [28], [29], [47]. The aim of this study was to test the influence of the NF-κB signaling pathway on the enhancer activity of the TSDR and thus on the maintenance of *Foxp3* expression. In agreement with previous publications [15], [28], we observed efficient nuclear localization of canonical NF-κB subunits upon T cell stimulation. However, the previously postulated NF-κB binding site within the TSDR [10] was not transcriptionally responsive to cell stimulation and NF-κB activation, implying that NF-κB did not regulate this site. Hence, it remains unclear which factor binds to and regulates this critical site.

Nonetheless, it was still conceivable that NF-κB was able to bind to other sites within the TSDR. We therefore assessed the overall influence of NF-κB signaling on TSDR enhancer activity. Canonical NF-κB signaling is regarded to be strictly dependent on IKKβ-mediated degradation of IκBα [15]. In this study, by using mutated versions of these key proteins, we could demonstrate that complete abrogation of NF-κB signaling did not considerably impair TSDR enhancer activity. Even though the lack of IKKβ kinase activity slightly weakened TSDR enhancer activity, the super-repressor did not show any such effect suggesting that IKKβ did not act via the classical NF-κB pathway. Instead, cross-reactivity of IKKβ to other signaling pathways [48] might have influenced TSDR enhancer activity. In a similar experiment, Long *et al.* showed that upon overexpression of the super-repressor in Jurkat cells (a human T cell line) TSDR enhancer activity is significantly reduced [25]. However, in their experiment the super-repressor also represses activity of the transcriptionally active *Foxp3* promoter. The *Foxp3* promoter construct used in our study does not have transcriptional activity on its own [10]. For this reason, we believe that in the study by Long *et al*. the observed reduction of the TSDR enhancer activity might be mediated by a compromised activity of the *Foxp3* promoter, whereas in our system we primarily examine effects on the TSDR. This interpretation is in line with the findings that c-Rel binds to the *Foxp3* promoter but not to the TSDR in TGFβ-induced and *ex vivo* isolated Tregs [29]. In conclusion, the data presented in this study argue that the classical NF-κB signaling pathway is not involved in the control of TSDR enhancer activity but may regulate other regulatory elements of the *Foxp3* gene locus, such as the *Foxp3* promoter or the pioneer element [30].

The NF-κB subunit c-Rel has been shown to have the most drastic impact on Treg development of all NF-κB family members. c-Rel^−/−^ Tregs (the few that do develop in c-Rel^−/−^ mice) express normal levels of *Foxp3* (this study and [24], [35]) and exhibit normal Treg transcriptional signature and suppressive capacity [35], indicating that an essential function of c-Rel emerges in developing Tregs, but not in mature Tregs. In accordance with this notion, we here show that c-Rel^−/−^ Tregs displayed stable *Foxp3* expression and a demethylated TSDR. Interestingly, c-Rel has the capacity to induce chromatin remodeling as it has been shown for the *Il2* locus and as it has been proposed for the *Foxp3* pioneer element [30], [36]. However, our data suggest that in developing Tregs c-Rel is not involved in TSDR demethylation. So far it is not known whether c-Rel or any other NF-κB subunit possess the ability to differentiate between methylated and unmethylated DNA, but as c-Rel has been detected to occupy the TSDR in Jurkat cells [25] harboring a fully methylated TSDR (unpublished data), binding of c-Rel to the TSDR most likely does not require DNA demethylation.

Viewed as a whole, the data presented in this study suggest that TSDR enhancer activity and its epigenetic fixation are controlled in a T cell-specific, but NF-κB-independent manner. The biological function of NF-κB binding to the TSDR as well as the identification of signaling pathways that ensure TSDR-mediated stability of *Foxp3* expression remain to be identified.

## Materials and Methods

### Mice

c-Rel-deficient mice (c-Rel^−/−^) [49], which were kindly provided by Alexander Visekruna (Institute for Medical Microbiology, Philipps University Marburg), and C57Bl/6 mice were bred in the animal facility of the Helmholtz Centre for Infection Research (Braunschweig). All mice were housed and handled under specific pathogen-free conditions in accordance with good animal practice as defined by FELASA and the national animal welfare body GV-SOLAS under supervision of the institutional animal welfare officer. Non-manipulated mice were euthanized by CO_2_ asphyxiation, and isolation of murine cells has been performed in compliance with the German animal protection law (TierSchG BGBI S. 1206; 18.05.2006). Number of animals used was notified to the Lower Saxony State Office for Consumer Protection and Food Safety according to the German laboratory animal reporting act (VersTierMeldV BGBl S. 2156; 04.11.1999).

### Cell Lines

The murine CD4^+^ T cell lymphoma cell line RLM-11 [50] was kindly provided by Marc Ehlers (Institute for Systemic Inflammation Research, Lübeck, Germany). RLM-11 cells were cultured in RPMI 1640 L-Glutamine medium (Invitrogen) supplemented with 10% FCS (Sigma-Aldrich), 50 U/ml penicillin, 50 U/ml streptomycin, 25 mM HEPES, 1 mM sodium pyruvate and 50 mM β-mercaptoethanol (all Biochrom). For stimulation, cells were treated with 10 ng/ml PMA and 500 ng/ml ionomycin (both Sigma-Aldrich). A20 cells [51] were kindly provided by Ingo Schmitz (HZI, Braunschweig, Germany) and cultured in RPMI 1640 L-Glutamine supplemented with 10% FCS, 50 U/ml penicillin, 50 U/ml streptomycin and 50 mM β-mercaptoethanol. RAW 264.7 cells [52], which were generously provided by Maximiliano Gutierrez (HZI, Braunschweig, Germany), were cultured in Dulbecco’s modified Eagle high glucose medium (Invitrogen) supplemented with 10% FCS and 2 mM L-Glutamine (Invitrogen). All cells were maintained at 37°C in a 5% CO_2_ atmosphere.

### Primary Cell Sorting and Cell Culture

Single cell suspensions obtained from isolated lymph nodes and spleens were subjected to surface staining using the according antibodies (CD4: clone RM4-5, BioLegend; CD62L: clone MEL-14, eBiosciences; CD25: clone PC61.5, BioLegend; CD8: clone 53-6.7, eBiosciences). Cell sorting was carried out on the BD FACS Aria II (BD Biosciences) and purity of isolated cell subsets was generally >97%. For activation and expansion, cells were placed in cell culture dishes (Nunc) which had been coated with 1 µg/ml anti-CD3 (clone 145-2C11, eBiosciences) and 1 µg/ml anti-CD28 (clone 37.51, eBiosciences) in PBS over night at 4°C. Cells were seeded in supplemented RPMI 1640 L-Glutamine medium containing 10 ng/ml (Tconv) or 50 ng/ml (Tregs) IL-2 (R&D Systems).

### Plasmids

pGL3 basic, pGL3-Promoter Vector (here referred to as SV40-Pro) and pGL3-Control Vector (here referred to as SV40Pro-SV40Enh) were purchased from Promega. The pNF-kB-Luc (here referred to as NF-κB responsive element; Agilent Technologies) was used to asses NF-κB activity. pGL3-TSDR-SV40 [9] and pGL3-TSDR-FoxPro, pGL3-FoxPro, pCpGL-EFPro and pCpGL-TSDR-EFPro [10] have been generated previously. pCpGL-tandem-EFPro was created by the introduction of five repetitive CTGGGCCTATCCGGCT sequence elements into pCpGL-EFPro upstream of the *EF* promoter (predicted NF-κB binding site underlined). Expression vectors for IKKα-KD and IKKβ-KD (both carrying D145N mutations) as well as IKK-CA (also named IKK-EE) were described before [33], [53]. The expression vector for the dominant negative IκBα (super-repressor) was reported before [34] and was generously provided by Dr. Maximiliano Gutierrez (HZI, Braunschweig, Germany).

### Luciferase Assay

All luciferase assays were performed as “dual luciferase assays” (Promega). 1×10^6^ RLM-11 cells were transfected with 1 µg of pGL3 firefly luciferase reporter vector incorporating the gene elements of interest and 0.2 µg of renilla luciferase vector pRL-TK, the latter one serving as internal control. Transfections were carried out in 100 µl transfection solution from the Cell Line Nucleofector Kit V in the Nucleofector 2b Device (Lonza) using program A-023. Cells were cultured in 1 ml supplemented RPMI 1640 L-Glutamine medium. After a resting period of 3 hrs, fresh medium was added to the cultures and cells were stimulated with 10 ng/ml PMA and 500 ng/ml ionomycin. For co-expression of recombinant proteins, plasmids encoding these proteins were co-transfected together with the luciferase plasmids. Transfection was carried out as described above. For each co-expressed recombinant protein, 1 µg expression vector was used. A20 cells were transfected the same way, with the difference that 2 µg of the desired firefly luciferase plasmid were transfected using program L-13. Transfection of RAW 264.7 cells was carried out with 2×10^6^ cells and 2 µg of pGL3 firefly luciferase reporter vector using nucleofector program D-032. Cells were cultured in 1.5 ml supplemented DMEM in a 12-well plate and stimulated the next day with 40 ng/ml IFN-γ and 100 ng/ml LPS (Sigma-Aldrich). Luciferase activities were measured 2–20 hrs later (RLM-11), 16–20 hrs later (A20) or 14 hrs later (RAW 264.7) using the Glomax instrument (Promega). For this, 50 µl of each firefly and renilla substrate were injected into 40–60 µl out of 100 µl cell lysate. Luciferase signals of firefly were normalized to the renilla luciferase signals and standard deviation of performed triplicates were calculated.

### Western Blot

In order to prepare cytoplasmic and nuclear extracts, cells were fractionized using the NE-PER Nuclear and Cytoplasmic Extraction Reagents supplemented with Halt Protease Inhibitors and Halt Phosphatase Inhibitors (Thermo Scientific). Proteins from extracts were separated by SDS-PAGE (Bio-Rad) and subsequently transferred to polyvinylidene fluorid membrane (GE Healthcare). Proteins of interest were detected using antibodies against p105/p50 (Epitomics), p100/p52 (R&D Systems), p65 and c-Rel as well as lamin B (Santa Cruz Biotechnology) and p44/p42 (Cell Signaling) in combination with horseradish peroxidase-conjugated secondary antibodies (Southern Biotechnolgy). Subsequently, chemiluminescence reactions were performed (ECL136II-system, Thermo Scientific). Exposed films were scanned and digitized images were analyzed using the software ImageJ 1.47 [54]. Here, protein signals were indexed by measuring their relative mean grey value per area.

### Flow Cytometric Analyses

Intracellular Foxp3 staining was carried out using the murine Foxp3 staining kit (eBiosciences). In order to exclude dead cells, cells were subjected to the live/dead fixable blue dead cell stain kit (Invitrogen). Flow cytometric measurements were accomplished on LSRII (BD Biosciences).

### Methylation Analysis by Pyrosequencing

Genomic DNA from cells of interest was obtained using the NucleoSpin Tissue kit (Macherey-Nagel). Genomic DNA was subjected to bisulfite conversion using the EZ DNA Methylation Kit (Zymo Research). The murine TSDR was amplified by PCR containing 100 ng of bisulfite-converted genomic DNA, HotStar Taq PCR buffer (Qiagen), 1 U HotStar Taq DNA polymerase, 2.5 mM MgCl2 and 0.38 µM each of TSDR-for (AAGGGGGTTTTAATATTTATGAGG) and TSDR-rev (CCTAAACTTAACCAAATTTTTCTACCA) primer in a final volume of 25 µl (Cycle: 95°C for 15 min; 45×95°C for 30 sec, 57°C for 1 min, 72°C for 1 min; 72°C for 7 min). The PCR product was analyzed by gel electrophoresis. The pyrosequencing procedure was performed on a Pyromark Q96 ID (Qiagen) according to the manufacturer’s protocol, including 40 µl of the PCR product, Pyromark Gold Q96 reagents (Qiagen), Pyromark buffers (Qiagen), Streptavidin Sepharose (GE Healthcare) and the sequencing primers TSDR1 (AACCAAATTTTTCTACCATTA), TSDR2 (AAAACAAATAATCTACCCC) or TSDR3 (AATAAACCCAAATAAAATAATATAAAT). The methylation rate was determined by the Pyromark Q96 software. A rate was excluded if the quality criteria (Pyromark Q96 standard settings) failed for that CpG motif. The methylation rate was translated into a color code as previously described [9].

## Supporting Information

Figure S1 **Activation of NF-kB family members in stimulated RLM-11 cells.** Films generated by western blotting described in Figure 2A were digitized by scanning, and specific antibody-mediated signals were analyzed by ImageJ. Normalization of the measured mean grey values per area for the cytoplasmic and nuclear proteins was achieved by using the p44/42 and laminB signals, respectively. The resulting relative values were plotted against the indicated time points after stimulation.
(EPS)

Figure S2 **Degradation of IκBα is not required for TSDR enhancer activity during the early phase of stimulation.** Luciferase plasmids integrating either the NF κB RE or TSDR-FoxPro were co-transfected with either an empty vector (EV) or with a vector encoding the super-repressor (SR), a non-degradable form of IκBα, into RLM 11 cells. Cells were harvested 2, 4, 6, 8, and 10 hours after stimulation with PMA/iono. Dual luciferase assays were performed and luciferase activities are shown as percent of the 6 hour empty vector control. Standard deviations of performed triplicates are shown. One representative out of three independent experiments is depicted.
(EPS)
